# Reduction of DNA damage repair efficiency and accumulation of residual damage following chronic UVB-irradiation of HaCaT cells

**DOI:** 10.1371/journal.pone.0283572

**Published:** 2023-04-07

**Authors:** Marie M. Dorr, Patrick J. Rochette

**Affiliations:** 1 Centre de Recherche du CHU de Québec–Université Laval, Axe Médecine Régénératrice, Hôpital du Saint-Sacrement, Québec, Canada; 2 Centre LOEX de l’Université Laval, Québec, Canada; 3 Université Laval, Faculté de Médecine, Département d’Ophtalmologie, Université Laval, Québec, Canada; Carleton University, CANADA

## Abstract

Absorption of ultraviolet radiation (UVR) by DNA leads to the predominant formation of cyclobutane pyrimidine dimers (CPD). Since those CPD are responsible for the driver mutations found in skin cancers, their efficient repair is critical. We previously showed that pre-stimulation of fibroblasts with chronic low doses of UVB (CLUV) increases CPD repair efficiency. Since skin cancers are not arising from dermal fibroblasts, this observation is not directly relevant to cutaneous carcinogenesis. We have now exposed HaCaT keratinocytes to a CLUV irradiation protocol to determine whether this pre-stimulation influences CPD removal rate. Similar to fibroblasts, CLUV treatment leads to the accumulation of residual CPD in keratinocytes, which are not repaired but rather tolerated and diluted through DNA replication. In contrast to fibroblasts, in keratinocytes we find that CLUV pre-treatment reduces CPD removal of newly generated damage without inducing a higher sensitivity to UVR-induced cell death. Using our experimental data, we derived a theoretical model to predict CPD induction, dilution and repair that occur in keratinocytes when chronically UVB-irradiated. Altogether, these results suggest that the accumulation of unrepaired CPD and the reduction in repair efficiency caused by chronic UVB exposure might lead to an increase in skin cancer driver mutations.

## Introduction

Exposure to solar light is the main risk factor for keratinocyte cancers (KC), the most common human neoplasia [1, 2]. The genotoxic effect of sunlight is attributed to DNA damage induced by ultraviolet radiation (UVR). Long UVB wavelengths (290–315 nm) are the main wavelengths responsible for skin cancer initiation and promotion [3, 4]. Indeed, the direct absorption of UVB photons by DNA leads to the generation of the two main types of UVR-induced DNA damage, *i*.*e*. cyclobutane pyrimidine dimers (CPD) and (6–4) pyrimidine-pyrimidone photoproducts (6-4PP) [5]. CPD are the most abundant (up to 85% of all UVR-induced DNA damage) and are highly mutagenic [3, 6]. They are responsible for the C → T transition mutations at dipyrimidine sites, the main mutation type found in sun-related skin cancers [7].

Skin cells harbor different mechanisms to avoid the conversion of UVB-induced CPD into skin cancer driver mutations, *i*.*e*. cell cycle arrest, DNA damage removal by nucleotide excision repair (NER) pathway and cell death by apoptosis [8]. Importance of NER for skin cancer prevention is well demonstrated by the fact that a deficiency in NER proteins, such as in xeroderma pigmentosum (XP) patients, leads to an increase of up to 2,000-fold in skin cancer occurrence [9]. NER efficiency has been extensively studied after a single acute UVB exposure [10–12]. However, the use of single irradiation is not representative of the human solar exposure, which is rather a multitude of low solar UV irradiations than a single acute one. The consequences of multiple exposures to UVR are still poorly understood, especially on epidermal cells. Only some studies by Vink *et al*. and Mitchell *et al*. [13–17] and more recently by our group [18–20] have used repeated UVB exposures to investigate the consequences of chronic irradiations on NER efficiency. Vink and Mitchell groups have shown that chronic irradiations lead to the formation and accumulation of unrepaired residuals CPD in epidermis and dermis. The group of Mitchell has also suggested that CPD repair is reduced as a consequence of the UVB stress [13–17]. Recently, we have demonstrated that a pre-stimulation of human diploid fibroblasts with chronic low doses of UVB (CLUV) leads to an accumulation of residual CPD but it was accompanied by an enhanced CPD repair efficiency [18–20].

Although relevant to decipher the mechanistic, human fibroblasts are not directly relevant to human cutaneous carcinogenesis. Indeed, KC are not arising from dermal fibroblasts but rather from keratinocytes. To our knowledge, chronic irradiation of human primary keratinocytes has not been done most likely because they cannot support the long-term confluency needed for chronic irradiation [19]. Indeed, primary diploid keratinocytes undergo differentiation followed by desquamation when they reach confluency [21]. However, the HaCaT human keratinocyte cell line tolerate long-term confluency.

In this study, we have thus exposed HaCaT cells to a chronic low dose of UVB (CLUV) protocol to evaluate the presence of CLUV-induced residuals CPD in human keratinocytes and to determine whether this pre-stimulation influences CPD removal rate. Our results show that the CLUV treatment leads to the accumulation of residuals CPD, that are not repaired but rather tolerated and diluted through DNA replication. We have found that CLUV pre-treatment reduces damage removal of newly generated CPD without inducing a higher sensitivity to UVR-induced cell death. Finally, using our experimental data, we derived a theoretical model to predict CPD induction, dilution and repair that occur in keratinocytes when chronically irradiated with UVB.

## Materials and methods

### Cell culture and UVB irradiation

Human immortalized keratinocytes (HaCaT) were cultured in Dulbecco’s modified Eagle’s medium (DMEM) (Wisent, Canada) containing 10% fetal bovine serum (FBS) (Wisent, Canada) and 1% antibiotics (10,000 units/mL penicillin and 10,000 μg/mL streptomycin) (Wisent, Canada) at 37°C with 8% CO_2_.

**Fig 1:**
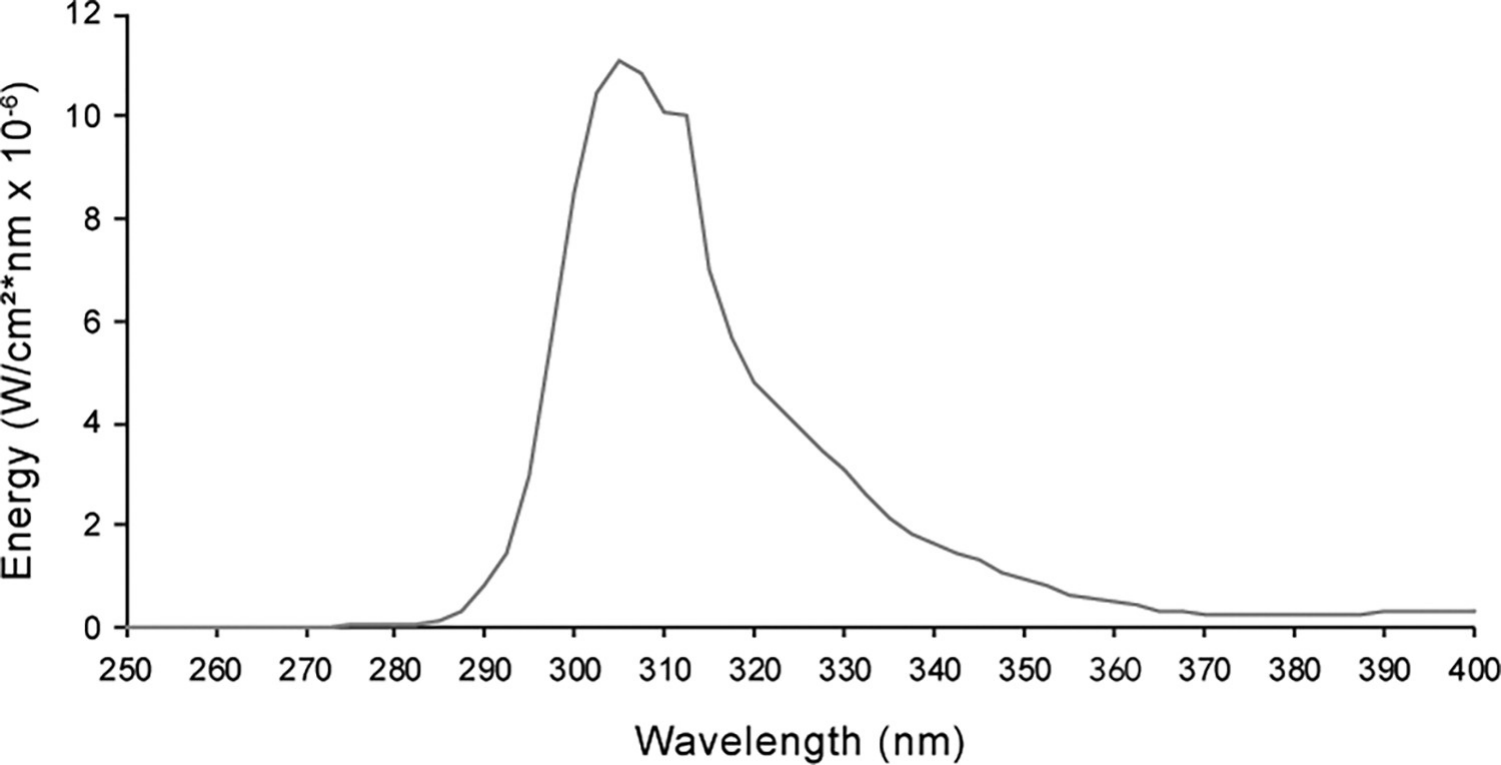
Emission spectrum of the UVR lamp (RPR-3000) after filtering through a Kodacel TA-407 clear 0.015” filter. The spectrum was derived from manufacturer’s specifications and was confirmed by our measurement using an International Light double monochromator spectroradiometer (IL7000 ⁄ 760D ⁄ 790).

Confluent HaCaT cells were irradiated at a dose rate of 19 J/m^2^ per sec using RPR-3000 lamps with a 300 nm emission peak (Southern New England Ultraviolet Co., USA) and a cellulose acetate sheet (Kodacel TA-407 clear 0.015 inches; Eastman-Kodak Co., USA) was used to filter out wavelengths below 295 nm (Fig 1). During irradiation, culture media was replaced with sterile phosphate buffered saline (PBS). The chronic irradiation protocol has been performed as previously described [19]. Briefly, the CLUV irradiation protocol consisted of 75 J/m^2^ UVB every 12 hours for 7.5 days (total: 15 irradiations, 1,125 J/m^2^). Twelve hours after the last irradiation of the CLUV protocol, cells were either harvested (CLUV) or exposed to a single acute irradiation (CLUV+Acute) of 400 J/m^2^ UVB for DNA repair analysis and 2,000 or 5,000 J/m^2^ for the cell viability assay. Prior to the acute irradiation in non-CLUV pre-stimulated cells, in order to mimic the CLUV experimental stress, culture media was changed for PBS at the same frequency than the CLUV cells. Twelve hours after the last irradiation from the CLUV treatment or directly after the acute irradiation is referred as T = 0.

### CPD detection

DNA was extracted using DNeasy Blood and Tissue Kit (Qiagen, Germany) according to the manufacturer’s instructions with an additional RNase treatment (final concentration 1.8 mg/ml). DNA concentration was measured by spectrophotometry (NanoDrop 2000; Thermoscientific, USA).

CPD detection was performed by immuno-slot blot as previously published [10] using anti-CPD (clone TDM2), dilution 1: 1,000 (Cosmo Bio Co., Japan) and rabbit anti-mouse HRP antibody, dilution 1: 5,000 (Jackson ImmunoResearch, USA). Blots analysis and quantification were performed with C-DiGit Blot Scanner and Image StudioLite software (LI-COR Biosciences, USA).

### Cellular replication analysis

Twelve hours following the last irradiation of the CLUV protocol or immediately after the acute irradiation, cells were harvested and reseeded at low density (9,500 cells/cm^2^) to allow replication. For the acute treatment, an irradiation of 250 J/m^2^ was used to induce the similar amount of CPD as found 12 h post-CLUV treatment. Cells were harvested and counted every 24 h for 7 days with a Z2 coulter counter (Beckman Coulter, USA).

### Cell viability analysis

Cell viability was determined by measuring metabolic activity using CellTiter 96® AQueous One Solution Cell Proliferation Assay (Promega, USA) according to the manufacturer’s protocol. Briefly, 24 h after a single acute irradiation of 2,000 or 5,000 J/m^2^ UVB, the culture media was removed and replaced with media supplemented with CellTiter 96® AQueous One Solution. Cells were incubated in the dark for 2 h at 37°C before recording the absorbance at 490 nm using a BioRad Microplate reader model 550 (BioRad Laboratories, USA). Non-irradiated cells were used as controls and their viability was considered as 100%.

## Results and discussion

### CLUV pre-stimulation of HaCaT leads to residual CPD accumulation and reduces CPD repair

**Fig 2:**
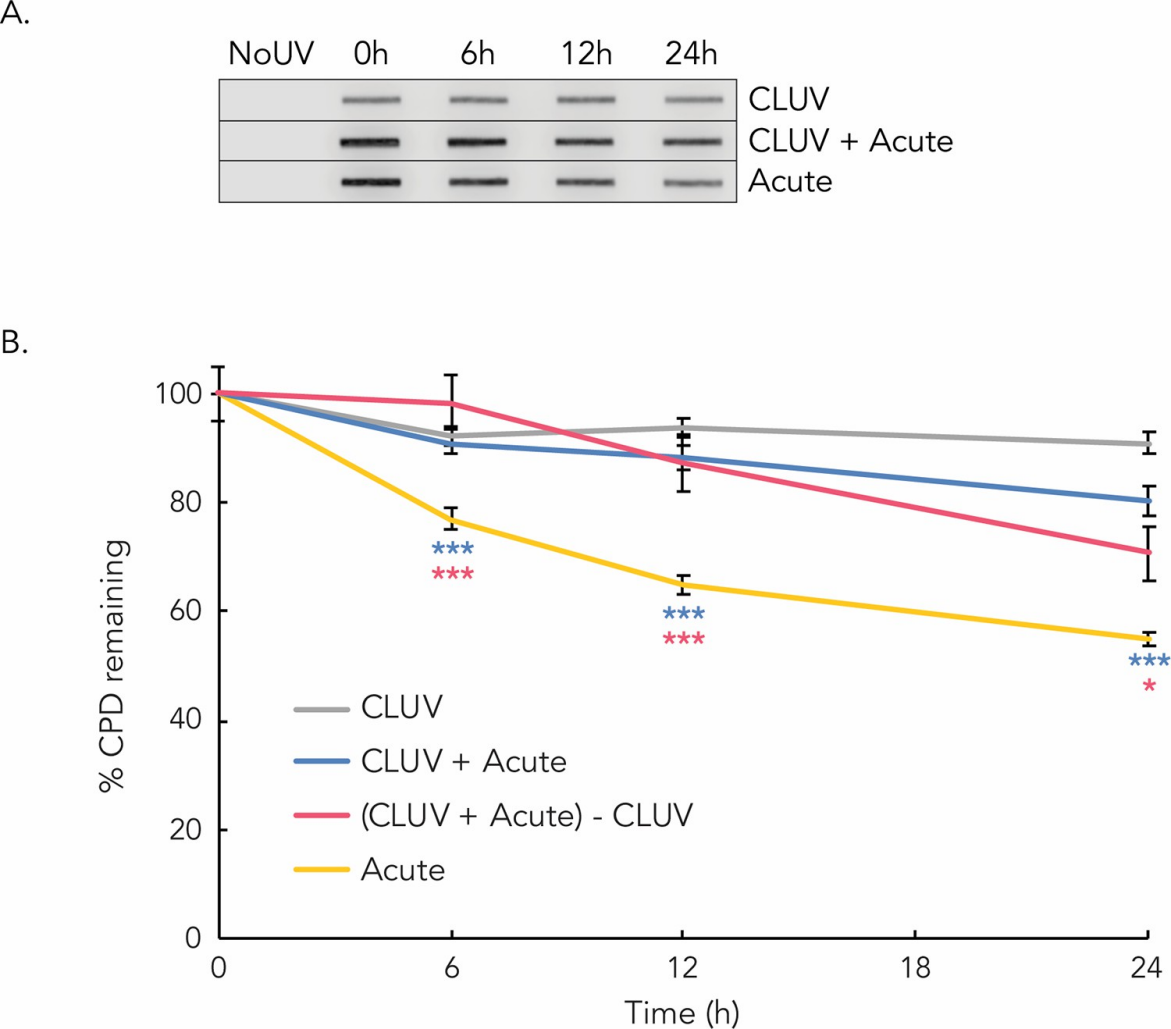
Repair kinetics of UVB-induced CPD in keratinocytes. Confluent keratinocytes were exposed to a chronic UVB-irradiation protocol (*i*.*e*. CLUV protocol; 75 J/m^2^ UVB, every 12 h, during 7.5 days for a total of 15 irradiations), an Acute irradiation (single exposure to 400 J/m^2^ UVB) or a combination of CLUV+Acute exposure. (**a**) The amount of UVR-induced CPD was assessed by immuno-slot-blot at 0, 6, 12 and 24 h using a specific anti-CPD antibody. Twelve hours after the last irradiation from the CLUV treatment or directly after the acute irradiation is referred as T = 0. Blot was cropped from different part of the same gel at the same exposure. (**b**) Quantification of the immuno-slot-blot was performed and repair kinetics were derived. Unirradiated cells (NoUV) were used as a negative control. CPD level at T = 0 was considered as 100% damage induced and the other time points were reported to T = 0. CLUV irradiation induces residual CPD that remain virtually unrepaired up to 36 h after the last irradiation. CPD induced by the acute UVB exposure are repaired efficiently leading to the removal of more than 45% of damage in 24 h. CPD induced by the CLUV pre-stimulation followed by the acute UVB irradiation (CLUV+Acute) are inefficiently repaired. Indeed, only 20% of CPD were removed in 24 h. To only evaluate the repair kinetic of the newly formed CPD induced by the Acute exposure in CLUV pre-stimulated cells, we subtracted the residual CPD from the CLUV treatment (i.e. (CLUV+Acute)-CLUV). CLUV pre-exposure significantly reduces the repair efficiency of newly generated CPD from the acute UVB irradiation. More precisely, at 24 h only 29% of acute UVB-induced CPD are repaired in CLUV pre-stimulated cells. Experiments were performed at least 7 times, in 2 independent CLUV protocols. Data presented as means ± SEM, (*** P≤0.001, * P≤0.05), Student T-Test. Blue asterisks represent the statistical comparison between “CLUV + Acute” and “Acute” conditions. Pink asterisks represent the statistical comparison between “(CLUV + Acute)–CLUV and “Acute” conditions.

We first examined whether a CLUV treatment could lead to the accumulation of residual damage and influence CPD repair efficiency in HaCaT. Twelve hours following the last irradiation of the CLUV protocol (referred as T = 0 in Fig 2), we can still detect CPD on the DNA. These are “unrepairable” residual CPD because they remain on DNA for at least 36 h following the last chronic irradiation (Fig 2). We further analyzed CPD removal in CLUV pre-stimulated cells (CLUV+Acute) and found that the repair of newly generated damage is significantly reduced in comparison to un-stimulated cells (Acute) (Fig 2). Indeed, 24 h post-acute irradiation, 20% of total CPD are repaired in CLUV pre-stimulated cells whereas 45% of damage are repaired when cells are not CLUV pre-stimulated. In the CLUV+Acute condition, cells contain residual CPD from the CLUV irradiation protocol but also newly generated damage from the single acute exposure to 400 J/m^2^ UVB. In order to evaluate the repair kinetic of the CPD induced by the acute exposure in the CLUV+Acute condition, we subtracted the signal of residual CPD from the CLUV treatment alone from the CLUV+Acute irradiation condition (*i*.*e*. (CLUV+Acute) − CLUV) as we have done previously [20]. We observe that the CLUV pre-treatment significantly reduced removal efficiency of newly generated CPD (Fig 2). Indeed, 24 h after the acute irradiation, only 29% of CPD are repaired in CLUV pre-stimulated cells compared to 45% in un-stimulated cells. Thus, CLUV pre-stimulation induces persistent damage and decreases newly formed CPD repair efficiency in keratinocytes.

The presence of chronic UVB-induced residual CPD in human keratinocytes is consistent with previous studies conducted *in vivo* in mouse epidermis and dermis and *in vitro* in normal diploid fibroblasts [14, 16, 18].

In this study, we also showed that pre-stimulation of keratinocytes with low UVB doses before an acute exposure significantly decreases the capacity to repair newly induced CPD. This supports the hypothesis proposed by Mitchell *et al*. stating that chronic exposure to UVB adversely affects CPD repair efficiency in mice [16]. Indeed, they observed a reduced CPD repair efficiency and a relatively high level of residual CPD 20 days following the last chronic irradiation, suggesting that CPD repair is reduced as a consequence of UVB stress.

In contrast with our results on HaCaT keratinocytes, using the exact same chronic irradiation protocol, our group recently showed that CPD repair is improved in chronically irradiated diploid dermal fibroblasts [20]. In healthy human skin, fibroblasts are mostly arrested in G0 and their replacement rate is considered low [22]. On the contrary, keratinocytes are dividing and gradually differentiating to eventually be removed by desquamation [23]. Slow repair rate in keratinocytes is counterintuitive given their fast replication rate. Indeed, conversion of mutagenic DNA damage, such as CPD, into skin cancer driver mutations depends on DNA replication [7]. Proper repair before cell division is therefore of great importance.

The cellular model used in this study is immortalized keratinocytes (HaCaT), harboring mutations in the gene coding for p53 [24]. P53 has been shown to regulate NER by transcriptionally activating XPC and DDB2 in fibroblasts [25–27]. Recently, we have shown that, in fibroblasts, CLUV pre-stimulation leads to an increase in p53, XPC and DDB2 gene and protein expressions [20]. However, response of keratinocytes to UVR has been shown to be distinct from other cell types, such as fibroblasts [11, 28]. More precisely, it has been reported that p53 status does not affect NER efficiency in keratinocytes [29–31]. Levels of XPC and DDB2 are affected in p53-deficient keratinocytes, but their basal levels are preserved, which seems sufficient for an efficient NER. This is true when cells are exposed to a single UV irradiation, not chronically. The fact that repair is slower after CLUV pre-stimulation suggests that chronic exposure depletes NER repair proteins. Cells can adapt by increasing their quantity of repair proteins, as we have observed with fibroblasts [20]. However, in a context where the cells are p53 deficient, they might not be able to adapt, and this results in a depletion of the proteins necessary to accomplish NER and a consequent reduction of CPD repair post-CLUV.

Normal UVR‐exposed human skin contains numerous patches of keratinocytes over‐expressing p53 long before carcinomas developed [32, 33]. The majority of these p53 clusters carry mutations in the p53 gene [33, 34]. These mutations are comparable to those found in squamous cell carcinomas, which leads to the hypothesis that p53 patches are precursors of skin cancers [34]. Using HaCaT cells harboring mutations in the p53 gene is therefore relevant to understand the impact of chronic irradiation in early precursors of skin cancers.

### Quantification of residual CPD induced by the CLUV treatment

**Fig 3:**
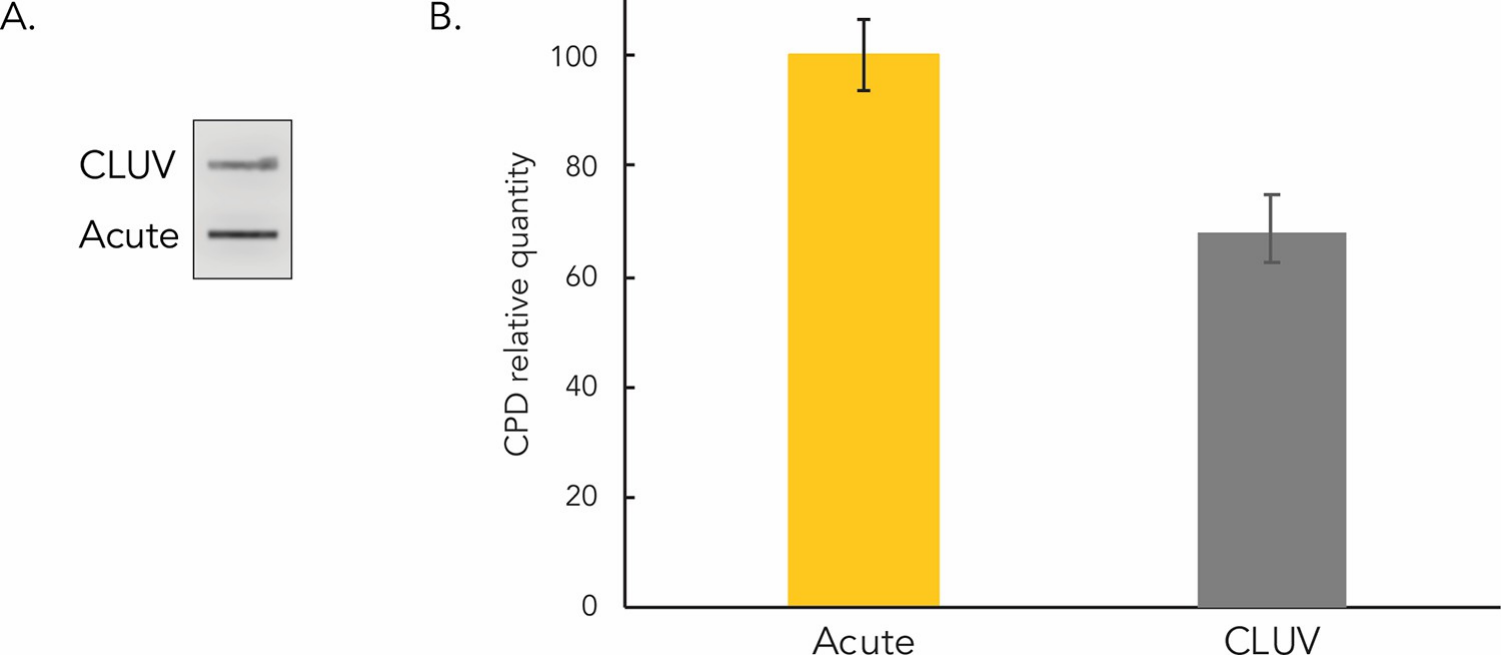
Quantification of residual CPD in CLUV irradiated keratinocytes. Keratinocytes were irradiated with the chronic CLUV protocol or with a single acute exposure to 400 J/m^2^ UVB. Cells were harvested and DNA was extracted immediately following the acute irradiation or 12 h after the last exposure of the CLUV treatment. (**a**) CPD were detected by immuno-slot-blot using a specific anti-CPD antibody. (**b**) Quantification of the immuno-slot-blot indicates that the signal level of CLUV-induced residual CPD is equivalent to 68% of those induced by a single exposure to 400 J/m^2^ UVB. Residual CPD induced by the CLUV are thus equivalent to the amount of CPD induced by approximately 273 J/m^2^. Since the cumulative UVB dose induced by the CLUV treatment is 1,125 J/m^2^, it can be estimated that 24% of the CLUV-induced CPD remain on the DNA 12h after the end of CLUV. Each experiment has been performed 5 times, in 2 independent CLUV protocols. Data presented as means ± SEM.

Residual CPD induced by the CLUV protocol were quantified 12 h after the last irradiation and compared to the amount of CPD induced immediately after a single exposure to 400 J/m^2^ of UVB. The signal level of residual CPD represents 68% of that induced by a single 400 J/m^2^ irradiation (Fig 3), which means there is the equivalent of 273 J/m^2^ of residual CPD. Since the cumulative dose induced by the CLUV is 1,125 J/m^2^, 273 J/m^2^ represents approximately 24% of the CLUV-induced CPD that remain on the DNA.

Some regions of the DNA are refractory to repair [35] and it has been shown that around 20% of UVR-induced CPD remain unrepairable [36]. We have previously analyzed residuals CPD induced by the same CLUV protocol as used in this project in dermal fibroblasts and we found an accumulation of 18% of residual CPD preferentially located on heterochromatin [18]. We found a higher (24% vs 18%) accumulation of CPD following a CLUV in keratinocytes, most likely due to the loss of CPD repair efficiency in keratinocytes compared the increase in repair efficiency in fibroblasts.

### Residual CPD dilution by DNA replication

**Fig 4:**
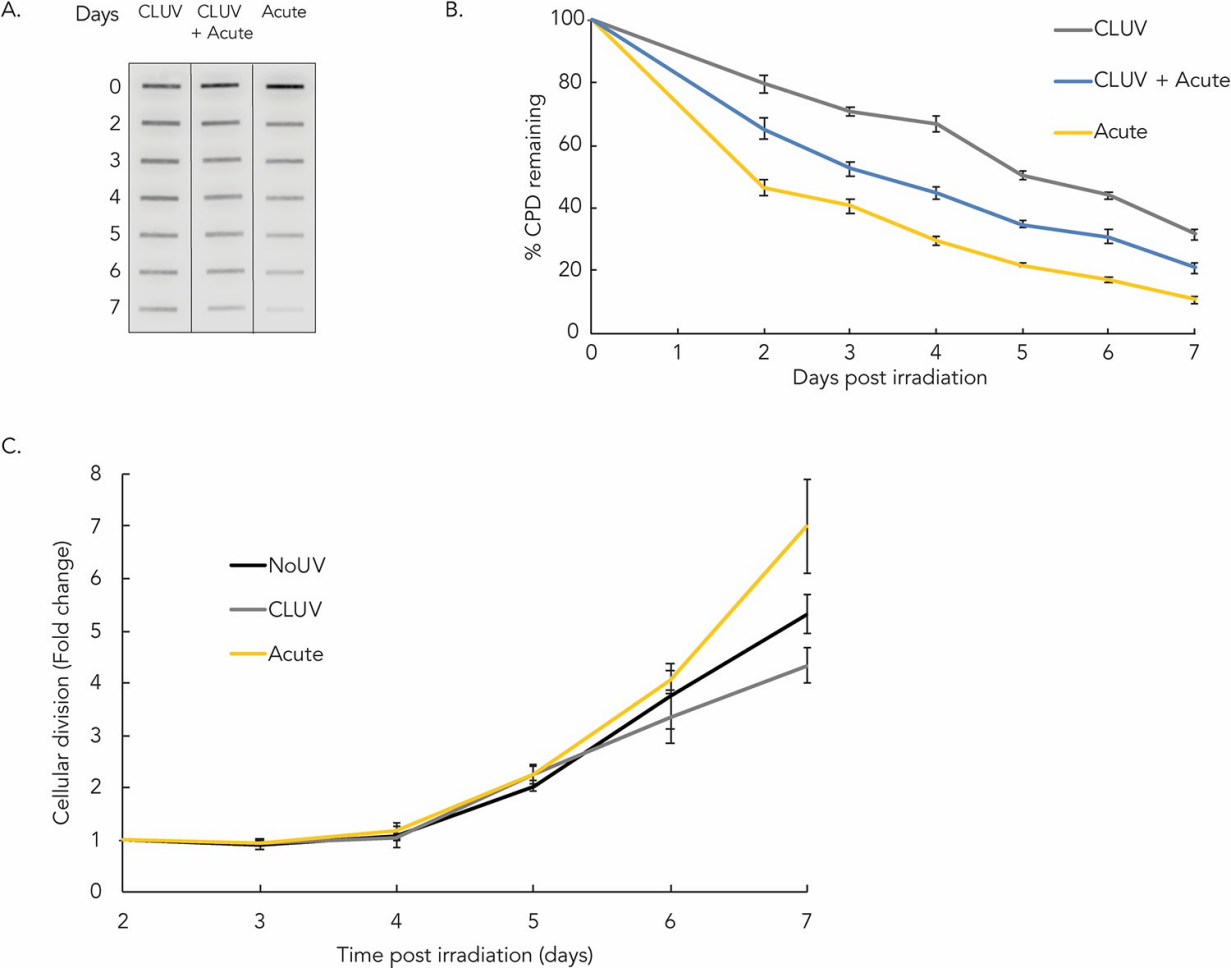
CPD fate after chronic UV exposure and lack of effect on proliferation of keratinocytes. Keratinocytes were irradiated with the chronic UVB irradiation protocol (CLUV), a single exposure to 400 J/m^2^ UVB (Acute) or a combination of CLUV+Acute exposure. (**a**) The amount of UVR-induced CPD was assessed by immuno-slot-blot using specific anti-CPD antibody at 0, 2, 3, 4, 5, 6 and 7 days post irradiation. Blot was cropped from different part of the same gel at the same exposure. (**b**) Quantification of the immuno-slot-blot was performed to determine the quantity of CPD. CPD level at T = 0 was considered as 100% damage induced and the other time points were reported to T = 0. From day 2 after the irradiation protocol until day 7, CPD level is reduced following a similar kinetics for all the irradiation conditions. Experiment was performed at least 3 times for each irradiation condition. (**c**) Cellular division was evaluated following different irradiation conditions. Keratinocytes were irradiated with the CLUV protocol or a single UVB dose of 250 J/m^2^ UVB (Acute) to induce a similar amount of CPD than the amount of residual CPD found following a CLUV treatment. Unirradiated keratinocytes (NoUV) were used as control. Twelve hours after the last irradiation of the CLUV treatment or directly after the acute irradiation, cells were reseeded at low density (9,500 cells/cm^2^) and counted every day for 7 days. The amount of cells at different time points was compared with the number of cells at day 2. Replication rate of CLUV- and Acute-irradiated cells was comparable to unirradiated cells. It takes approximately 5 days to reach a population doubling in all conditions. Experiment was performed in triplicate. Data presented as means ± SEM.

We investigated the fate of the CPD in HaCaT keratinocytes from day 2 to day 7 following the last irradiation to examine whether the removal of these damage was delayed in some irradiation conditions (Fig 4A and 4B). Regardless of the type of UVR treatment (CLUV, CLUV+Acute and Acute), 48 h following the last irradiation, damage are removed or diluted with a similar efficiency. Indeed, we observed that the percentages of remaining CPD, from day 2 to day 7, from the 3 different irradiation protocols were decreasing following a very similar slope.

We further determined whether HaCaT keratinocytes were able to replicate after an Acute or CLUV irradiation protocol. After a single acute irradiation of 250 J/m^2^ (Acute) or a CLUV treatment, we reseeded the cells at low density and we counted them every day to determine cellular replication rate (Fig 4C). CLUV- and Acute-irradiated cells as well as the unirradiated control replicate all at a similar rate. Indeed, it takes approximately 5 days to reach a population doubling in all conditions. We can observe a faster proliferation rate in the Acute condition and a slower one in the CLUV condition at day 7, but it is not statistically significant. This indicates that the CLUV protocol and, by association, residual CPD do not affect the cell capacity to proliferate.

We have previously shown that CLUV-induced residual CPD can be diluted through cellular division via semiconservative DNA replication in dermal fibroblasts [18]. CLUV-treated human fibroblasts have shown a slower replication rate than non-irradiated cells. In human keratinocytes, we did not find any differences in replication rate when cells were exposed to a CLUV or Acute irradiation. This indicates that the CPD decrease we observed from day 2 to 7 is most likely due to damage dilution occurring with cellular division rather than delayed repair. Indeed, the fact that cells irradiated with different protocols harbor the same replication capacity is consistent with a similar CPD dilution in each UVR irradiation protocol.

### CLUV-irradiation of HaCaT does not affect their sensitivity to UVR-induced cell death

**Fig 5:**
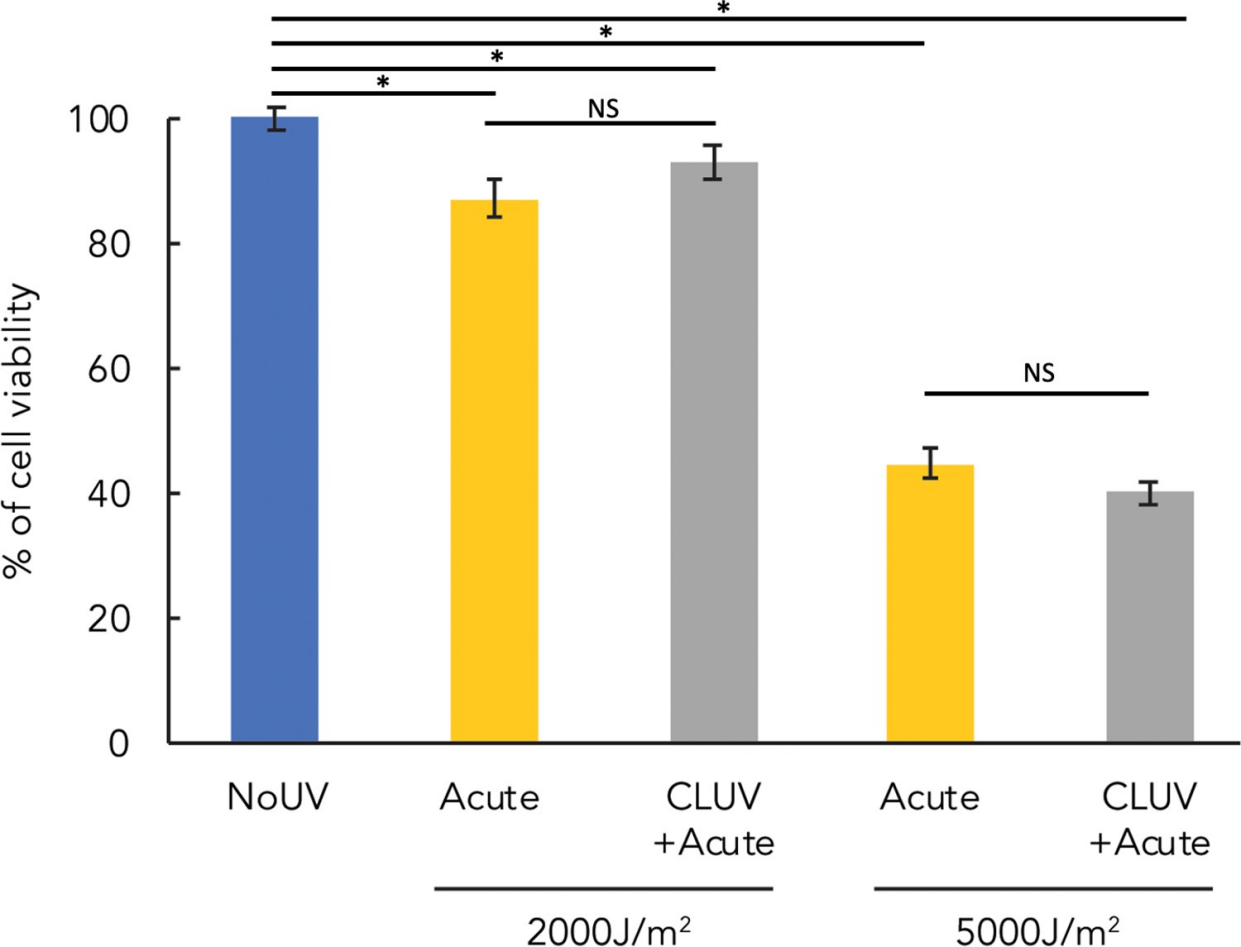
Sensitivity to UVB-induced cell death after a CLUV pre-stimulation. Keratinocytes pre-stimulated or not with the CLUV treatment were irradiated with an acute lethal exposure of 2,000 J/m^2^ or 5,000 J/m^2^ of UVB. Cellular viability was assessed 24h after the acute irradiation using a metabolic assay. Unirradiated cells (NoUV) were used as a negative control and their viability was considered as 100%. CLUV pre-stimulation of keratinocytes does not lead to an increase in UVB-induced cell death sensitivity. Experiment was performed 8 times per condition. Data presented as means ± SEM, (* P≤0.05, NS P>0.05), Student T-Test.

Programmed cell death is a mechanism used by keratinocytes to avoid the conversion of DNA damage into mutation [37]. By using an assay measuring cellular metabolism, we investigated whether CLUV pre-stimulation influences cell death sensitivity in HaCaT keratinocytes irradiated with an acute lethal dose of 2,000 or 5,000 J/m^2^ of UVB (Fig 5). We found no difference in cell death sensitivity between keratinocytes pre-stimulated or not with a CLUV treatment.

Unrepaired DNA damage have been shown to be a signal for cell death [38] and more precisely for apoptosis [39]. However, we found that a CLUV pre-stimulation and the residual CPD induced by this chronic irradiation have no influence on keratinocyte sensitivity to UVB-induced cell death. This is consistent with a previous study done by our group showing no difference in UVR-induced cell death sensitivity following a CLUV pre-stimulation in human primary fibroblasts [19]. The amount of residual CPD induced by the chronic irradiation, *i*.*e*. equivalent to 273 J/m^2^ UVB, is minimal compared to the amount induced by the UVB doses used to induce cell death (2,000 and 5,000 J/m^2^). We thus hypothesized that the amount of CLUV-induced residual CPD is insufficient to have an effect in cell death sensitivity.

### Theoretical model for CPD removal by dilution and repair during a chronic UVB irradiation

**Fig 6:**
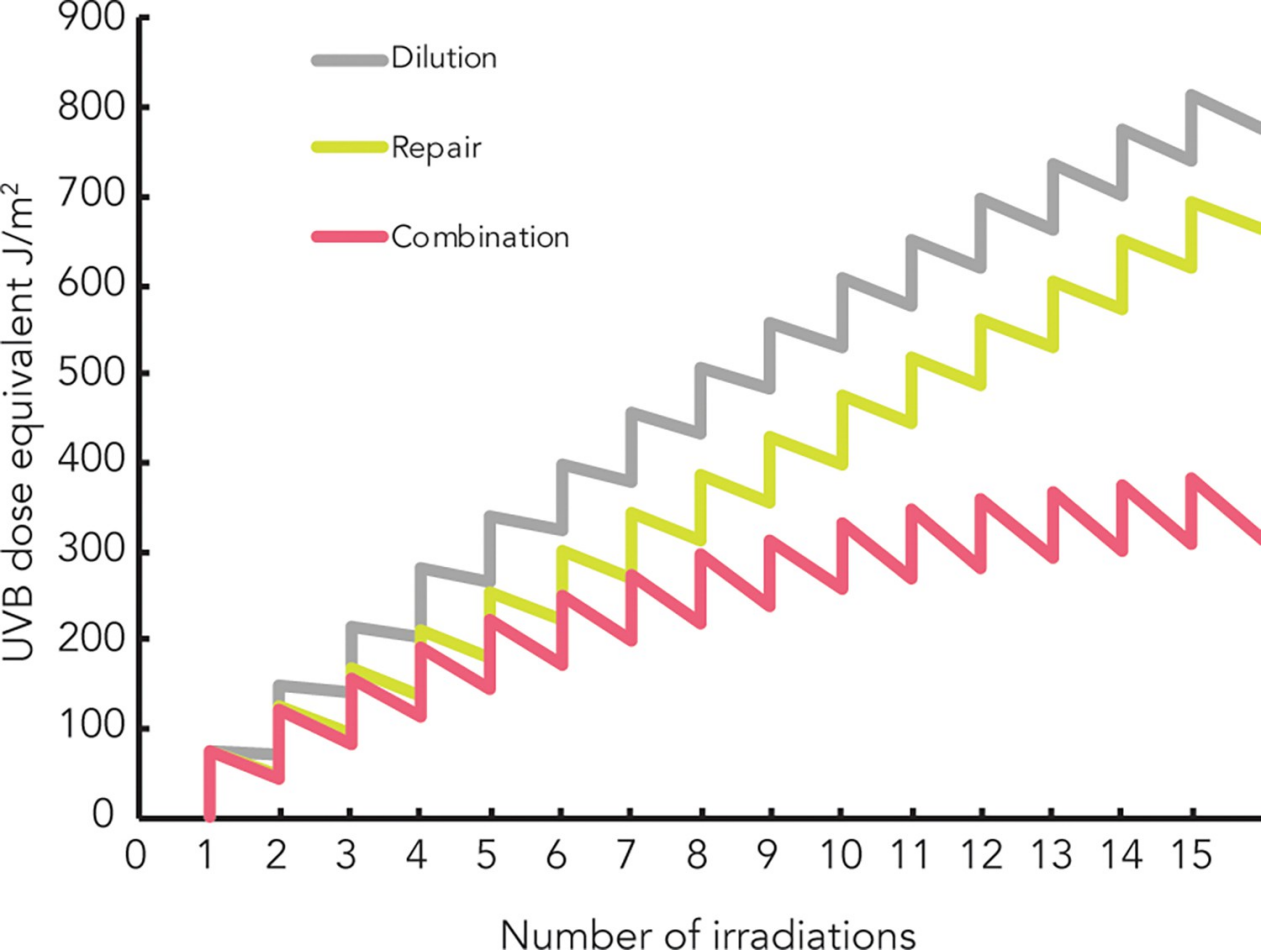
Theoretical model for CPD removal by dilution and repair during a chronic UVB irradiation. We derived a model to predict the amount of CPD, expressed in UVB dose equivalent (J/m^2^), that remains on DNA during a chronic irradiation. The model take into account the repair and the dilution of CPD after each chronic irradiation. Each individual irradiation from the CLUV protocol leads to an induction of CPD, represented by an increase of 75 J/m^2^ UVB equivalent. **Repair** of the newly induced CPD after each CLUV irradiation was modeled using the acute repair efficiencies we measured between 0 h-12 h (35.3% of new damage repaired) and 12 h-24 h (9.8% of remaining damage) (green line). Rate of CPD removal by **dilution** was determined using the equation of the regression line for the CLUV treatment (y = -0.4012x + 100.53). It was applied to all the remaining damage (grey line). CPD reduction from DNA repair and dilution were combined (red line). Using this model, we determined that the theoretical amount of CPD induced by the chronic irradiation represents approximately the level induced by 312 J/m^2^ of UVB. This corresponds to 28% of the CLUV-induced CPD that persist on the DNA. We found that the actual measured amount induced by the CLUV protocol is similar (273 J/m^2^), validating our model.

In this study, we have derived the repair and dilution rates of CPD following a chronic UVB irradiation in HaCaT (Figs 2 and 4B). Using these kinetics, we were able to derive a theoretical model to predict CPD induction and removal occurring during a chronic irradiation. In this model, CPD level is expressed in UVB dose equivalent (in J/m^2^) and each of 15 individual irradiation leads to CPD formation, represented by a peak in damage induction. Two main parameters to consider to determine CPD removal following UVR irradiation are the repair and the dilution rates. For the repair rate, we considered each UVB irradiation of the CLUV protocol as a small acute exposure and we thus used the acute repair kinetics as depicted in Fig 2 between 0 h-12 h (removal of 35.3% of the newly induced CPD) and 12 h-24 h (removal of 9.8% of the remaining CPD). This allowed us to estimate CPD repair occurring after each of the chronic UVB irradiation of the CLUV protocol (Fig 6; downward segment of the green curve). To derive dilution portion of the CPD removal we used the regression line for CLUV treated cells in Fig 3B (equation of the regression line is y = -0.4012x + 100.53). We used this equation to predict the dilution rate of CPD between each irradiation of the CLUV treatment (Fig 6; downward segment of the grey curve). The dilution rate was applied to all the remaining CPD and not only to the newly generated CPD as for the repair rate. Since both dilution and repair occur between each irradiation of the CLUV protocol, we combined their kinetics and thus subtracted the diluted and the repaired damage after every individual irradiation (Fig 6; downward segment of the red curve). When combining equations of CPD dilution and repair during the course of CLUV irradiation protocol, we found that the remaining amount of CPD should theoretically be around the equivalent of those induced by a single UVB irradiation of 312 J/m^2^. This represents 28% of the CLUV-induced CPD that remain on the DNA, be approximately the 24% (equivalent to 273 J/m^2^) remaining CPD we found experimentally when measuring residual CPD (Fig 3).

The fact that the measured residual CPD induced by the CLUV protocol (Fig 3) is approximately the same amount of damage that the one found using our model is a proof that the theoretical model is accurate and that both dilution and repair are involved in CPD removal during a CLUV treatment.

## Conclusion

We showed that chronic UVB irradiations of HaCaT keratinocytes lead to a reduction of CPD repair efficiency and to the accumulation of unrepairable damage. Those residual CPD are tolerated by the cells and do not alter their proliferative capacity or their cell death sensitivity. Using the measured repair kinetics and dilution curve of UVB-induced CPD, we have been able to derive a theoretical model to predict the induction, dilution and repair of these DNA damage during a chronic UVB irradiation. This reduction in excision repair and the accumulation of residual CPD in the epidermis suggest that chronic low-dose exposure to sunlight might favor skin carcinogenesis.

## Supporting information

S1 Data (XLSX)

S1 Raw images (PDF)

## References

[1] American Cancer Society AC. Key Statistics for Basal and Squamous Cell Skin Cancers:; 2018. https://www.cancer.org/cancer/basal-and-squamous-cell-skin-cancer/about/key-statistics.html.

[2] Urbach F. Ultraviolet radiation and skin cancer of humans. J Photochem Photobiol B. 1997;40(1):3–7. Epub 1997/08/01. doi: 10.1016/s1011-1344(97)00029-8 . 9301039

[3] Besaratinia A, Yoon JI, Schroeder C, Bradforth SE, Cockburn M, Pfeifer GP. Wavelength dependence of ultraviolet radiation-induced DNA damage as determined by laser irradiation suggests that cyclobutane pyrimidine dimers are the principal DNA lesions produced by terrestrial sunlight. FASEB J. 2011;25(9):3079–91. Epub 2011/05/27. doi: 10.1096/fj.11-187336 ; PubMed Central PMCID: PMC3157686. 21613571 PMC3157686

[4] Nishisgori C. Current concept of photocarcinogenesis. Photochemical & photobiological sciences: Official journal of the European Photochemistry Association and the European Society for Photobiology. 2015;14(9):1713–21. Epub 2015/07/16. doi: 10.1039/c5pp00185d . 26177397

[5] Sage E. Distribution and repair of photolesions in DNA: genetic consequences and the role of sequence context. Photochem Photobiol. 1993;57(1):163–74. Epub 1993/01/01. doi: 10.1111/j.1751-1097.1993.tb02273.x . 8389052

[6] You YH, Lee DH, Yoon JH, Nakajima S, Yasui A, Pfeifer GP. Cyclobutane pyrimidine dimers are responsible for the vast majority of mutations induced by UVB irradiation in mammalian cells. J Biol Chem. 2001;276(48):44688–94. Epub 2001/09/27. doi: 10.1074/jbc.M107696200 . 11572873

[7] Brash DE. UV signature mutations. Photochem Photobiol. 2015;91(1):15–26. doi: 10.1111/php.12377 ; PubMed Central PMCID: PMC4294947. 25354245 PMC4294947

[8] Sancar A, Lindsey-Boltz LA, Unsal-Kacmaz K, Linn S. Molecular mechanisms of mammalian DNA repair and the DNA damage checkpoints. Annu Rev Biochem. 2004;73:39–85. Epub 2004/06/11. doi: 10.1146/annurev.biochem.73.011303.073723 . 15189136

[9] Cleaver JE, Lam ET, Revet I. Disorders of nucleotide excision repair: the genetic and molecular basis of heterogeneity. Nat Rev Genet. 2009;10(11):756–68. Epub 2009/10/08. doi: 10.1038/nrg2663 . 19809470

[10] Mallet JD, Dorr MM, Drigeard Desgarnier MC, Bastien N, Gendron SP, Rochette PJ. Faster DNA Repair of Ultraviolet-Induced Cyclobutane Pyrimidine Dimers and Lower Sensitivity to Apoptosis in Human Corneal Epithelial Cells than in Epidermal Keratinocytes. PloS one. 2016;11(9):e0162212. doi: 10.1371/journal.pone.0162212 . 27611318 PMC5017652

[11] D’Errico M, Teson M, Calcagnile A, Proietti De Santis L, Nikaido O, Botta E, et al. Apoptosis and efficient repair of DNA damage protect human keratinocytes against UVB. Cell Death Differ. 2003;10(6):754–6. Epub 2003/05/23. doi: 10.1038/sj.cdd.4401224 . 12761584

[12] Fernandez TL, Van Lonkhuyzen DR, Dawson RA, Kimlin MG, Upton Z. In vitro investigations on the effect of dermal fibroblasts on keratinocyte responses to ultraviolet B radiation. Photochem Photobiol. 2014;90(6):1332–9. doi: 10.1111/php.12317 . 25039640

[13] Vink AA, Berg RJ, de Gruijl FR, Lohman PH, Roza L, Baan RA. Detection of thymine dimers in suprabasal and basal cells of chronically UV-B exposed hairless mice. J Invest Dermatol. 1993;100(6):795–9. Epub 1993/06/01. doi: 10.1111/1523-1747.ep12476606 . 7684426

[14] Vink AA, Berg RJ, de Gruijl FR, Roza L, Baan RA. Induction, repair and accumulation of thymine dimers in the skin of UV-B-irradiated hairless mice. Carcinogenesis. 1991;12(5):861–4. Epub 1991/05/01. doi: 10.1093/carcin/12.5.861 . 2029750

[15] Mitchell DL, Byrom M, Chiarello S, Lowery MG. Effects of chronic exposure to ultraviolet B radiation on DNA repair in the dermis and epidermis of the hairless mouse. J Invest Dermatol. 2001;116(2):209–15. doi: 10.1046/j.1523-1747.2001.01192.x . 11179995

[16] Mitchell DL, Greinert R, de Gruijl FR, Guikers KL, Breitbart EW, Byrom M, et al. Effects of chronic low-dose ultraviolet B radiation on DNA damage and repair in mouse skin. Cancer Res. 1999;59(12):2875–84. . 10383149

[17] Mitchell DL, Volkmer B, Breitbart EW, Byrom M, Lowery MG, Greinert R. Identification of a non-dividing subpopulation of mouse and human epidermal cells exhibiting high levels of persistent ultraviolet photodamage. J Invest Dermatol. 2001;117(3):590–5. doi: 10.1046/j.0022-202x.2001.01442.x . 11564164

[18] Berube R, Drigeard Desgarnier MC, Douki T, Lechasseur A, Rochette PJ. Persistence and Tolerance of DNA Damage Induced by Chronic UVB Irradiation of the Human Genome. J Invest Dermatol. 2018;138(2):405–12. doi: 10.1016/j.jid.2017.08.044 . 28951242

[19] Drigeard Desgarnier MC, Fournier F, Droit A, Rochette PJ. Influence of a pre-stimulation with chronic low-dose UVB on stress response mechanisms in human skin fibroblasts. PloS one. 2017;12(3):e0173740. doi: 10.1371/journal.pone.0173740 ; PubMed Central PMCID: PMC5354420. 28301513 PMC5354420

[20] Drigeard Desgarnier MC, Rochette PJ. Enhancement of UVB-induced DNA damage repair after a chronic low-dose UVB pre-stimulation. DNA Repair (Amst). 2018;63:56–62. Epub 2018/02/16. doi: 10.1016/j.dnarep.2018.01.008 . 29448173

[21] Sun TT, Green H. Differentiation of the epidermal keratinocyte in cell culture: formation of the cornified envelope. Cell. 1976;9(4 Pt 1):511–21. Epub 1976/12/01. doi: 10.1016/0092-8674(76)90033-7 . 1009573

[22] Yegorov YE, Zelenin AV. Duration of senescent cell survival in vitro as a characteristic of organism longevity, an additional to the proliferative potential of fibroblasts. FEBS Lett. 2003;541(1–3):6–10. Epub 2003/04/23. doi: 10.1016/s0014-5793(03)00298-9 . 12706810

[23] Fuchs E, Raghavan S. Getting under the skin of epidermal morphogenesis. Nat Rev Genet. 2002;3(3):199–209. Epub 2002/04/25. doi: 10.1038/nrg758 . 11972157

[24] Lehman TA, Modali R, Boukamp P, Stanek J, Bennett WP, Welsh JA, et al. p53 mutations in human immortalized epithelial cell lines. Carcinogenesis. 1993;14(5):833–9. Epub 1993/05/01. doi: 10.1093/carcin/14.5.833 . 8504475

[25] Hwang BJ, Ford JM, Hanawalt PC, Chu G. Expression of the p48 xeroderma pigmentosum gene is p53-dependent and is involved in global genomic repair. Proc Natl Acad Sci U S A. 1999;96(2):424–8. Epub 1999/01/20. doi: 10.1073/pnas.96.2.424 ; PubMed Central PMCID: PMC15152. 9892649 PMC15152

[26] Ford JM. Regulation of DNA damage recognition and nucleotide excision repair: another role for p53. Mutat Res. 2005;577(1–2):195–202. doi: 10.1016/j.mrfmmm.2005.04.005 . 15927209

[27] Therrien JP, Drouin R, Baril C, Drobetsky EA. Human cells compromised for p53 function exhibit defective global and transcription-coupled nucleotide excision repair, whereas cells compromised for pRb function are defective only in global repair. Proceedings of the National Academy of Sciences of the United States of America. 1999;96(26):15038–43. doi: 10.1073/pnas.96.26.15038 ; PubMed Central PMCID: PMC24769. 10611334 PMC24769

[28] D’Errico M, Teson M, Calcagnile A, Nardo T, De Luca N, Lazzari C, et al. Differential role of transcription-coupled repair in UVB-induced response of human fibroblasts and keratinocytes. Cancer Res. 2005;65(2):432–8. Epub 2005/02/08. . 15695384

[29] Ferguson BE, Oh DH. Proficient global nucleotide excision repair in human keratinocytes but not in fibroblasts deficient in p53. Cancer Res. 2005;65(19):8723–9. Epub 2005/10/06. doi: 10.1158/0008-5472.CAN-05-1457 . 16204041

[30] Ferguson-Yates BE, Li H, Dong TK, Hsiao JL, Oh DH. Impaired repair of cyclobutane pyrimidine dimers in human keratinocytes deficient in p53 and p63. Carcinogenesis. 2008;29(1):70–5. Epub 2007/11/07. doi: 10.1093/carcin/bgm244 ; PubMed Central PMCID: PMC4061977. 17984111 PMC4061977

[31] Oh DH, Yeh K. Differentiating human keratinocytes are deficient in p53 but retain global nucleotide excision repair following ultraviolet radiation. DNA Repair (Amst). 2005;4(10):1149–59. Epub 2005/07/27. doi: 10.1016/j.dnarep.2005.06.004 . 16043423

[32] Berg RJ, van Kranen HJ, Rebel HG, de Vries A, van Vloten WA, Van Kreijl CF, et al. Early p53 alterations in mouse skin carcinogenesis by UVB radiation: immunohistochemical detection of mutant p53 protein in clusters of preneoplastic epidermal cells. Proc Natl Acad Sci U S A. 1996;93(1):274–8. Epub 1996/01/09. doi: 10.1073/pnas.93.1.274 ; PubMed Central PMCID: PMC40221. 8552621 PMC40221

[33] Jonason AS, Kunala S, Price GJ, Restifo RJ, Spinelli HM, Persing JA, et al. Frequent clones of p53-mutated keratinocytes in normal human skin. Proceedings of the National Academy of Sciences of the United States of America. 1996;93(24):14025–9. doi: 10.1073/pnas.93.24.14025 ; PubMed Central PMCID: PMC19488. 8943054 PMC19488

[34] Rebel H, Mosnier LO, Berg RJ, Westerman-de Vries A, van Steeg H, van Kranen HJ, et al. Early p53-positive foci as indicators of tumor risk in ultraviolet-exposed hairless mice: kinetics of induction, effects of DNA repair deficiency, and p53 heterozygosity. Cancer Res. 2001;61(3):977–83. Epub 2001/02/28. . 11221893

[35] Rochette PJ, Brash DE. Human telomeres are hypersensitive to UV-induced DNA Damage and refractory to repair. PLoS genetics. 2010;6(4):e1000926. doi: 10.1371/journal.pgen.1000926 ; PubMed Central PMCID: PMC2861706. 20442874 PMC2861706

[36] Courdavault S, Baudouin C, Sauvaigo S, Mouret S, Candeias S, Charveron M, et al. Unrepaired cyclobutane pyrimidine dimers do not prevent proliferation of UV-B-irradiated cultured human fibroblasts. Photochem Photobiol. 2004;79(2):145–51. doi: 10.1562/0031-8655(2004)079&lt;0145:ucpddn&gt;2.0.co;2 . 15068027

[37] Kulms D, Schwarz T. Molecular mechanisms of UV-induced apoptosis. Photodermatol Photoimmunol Photomed. 2000;16(5):195–201. Epub 2000/11/09. doi: 10.1034/j.1600-0781.2000.160501.x . 11068857

[38] Roos WP, Thomas AD, Kaina B. DNA damage and the balance between survival and death in cancer biology. Nat Rev Cancer. 2016;16(1):20–33. Epub 2015/12/19. doi: 10.1038/nrc.2015.2 . 26678314

[39] Kulms D, Schwarz T. Molecular mechanisms involved in UV-induced apoptotic cell death. Skin Pharmacol Appl Skin Physiol. 2002;15(5):342–7. Epub 2002/09/20. doi: 10.1159/000064539 . 12239429

