# Supplementary material for: Reduction of DNA damage repair efficiency and accumulation of residual damage following chronic UVB-irradiation of HaCaT cells
Source: PLoS One. 2023 Apr 7;18(4):e0283572. doi: 10.1371/journal.pone.0283572 (PMC10081739; doi:10.1371/journal.pone.0283572)
Supplement: S1 Raw images — (PDF) [file pone.0283572.s002.pdf]

Raw blot for figure panel 2a. It is a slot blot, only the loaded samples show signal and there are no molecular weight markers. Image has been captured using Image Studio from LI-COR. Samples were loaded twice in case there is a problem with a sample (e.g. leakage in the well as seen in the CLUV + Acute top samples). We chose one of the two series for the final figure.

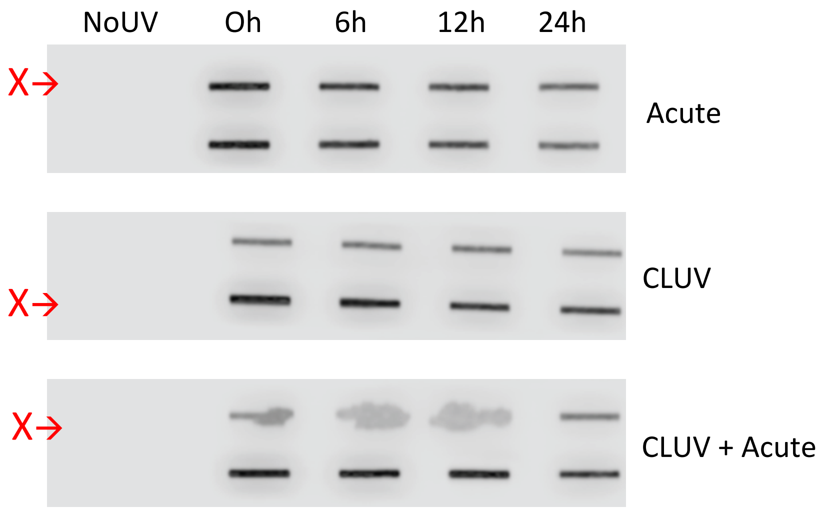

Raw blot for figure panel 3a. It is a slot blot, only the loaded samples show signal and there are no molecular weight markers. Image has been captured using Image Studio from LI-COR. Each of the 5 replicates (R1, R2, …) have been loaded twice in case there is a problem with a sample (e.g. leakage in the well). We chose one replicate for the final figure (framed).

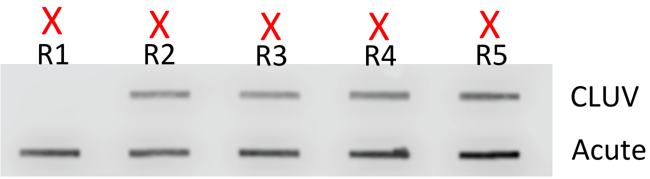

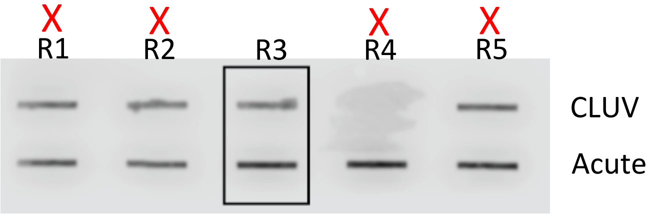

Raw blot for figure panel 4a. It is a slot blot, only the loaded samples show signal and there are no molecular weight markers. Image has been captured using Image Studio from LI-COR. We chose one replicate for the final figure (framed).

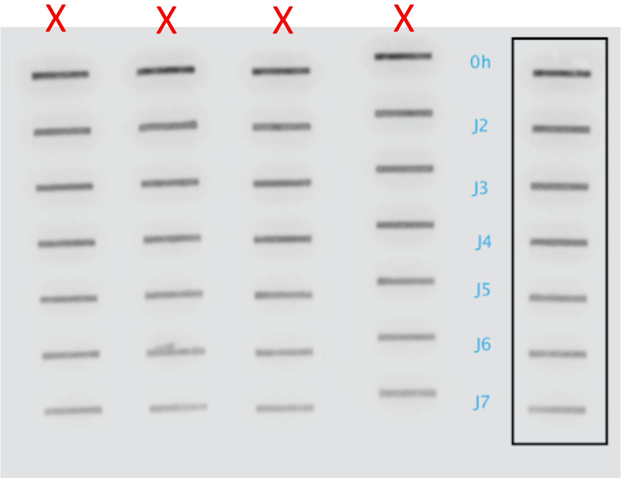

CLUV

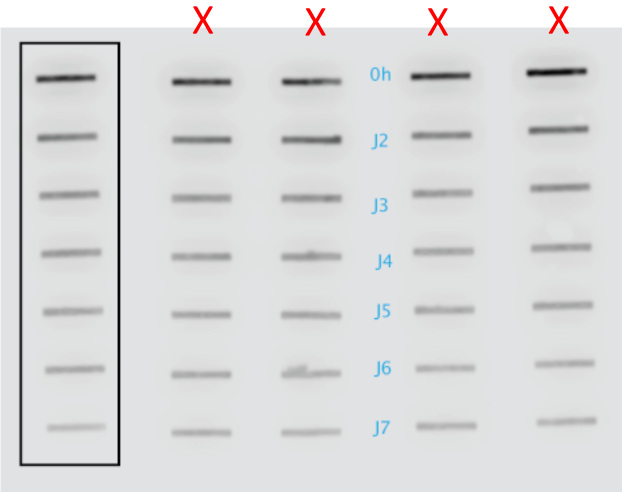

CLUV + Acute

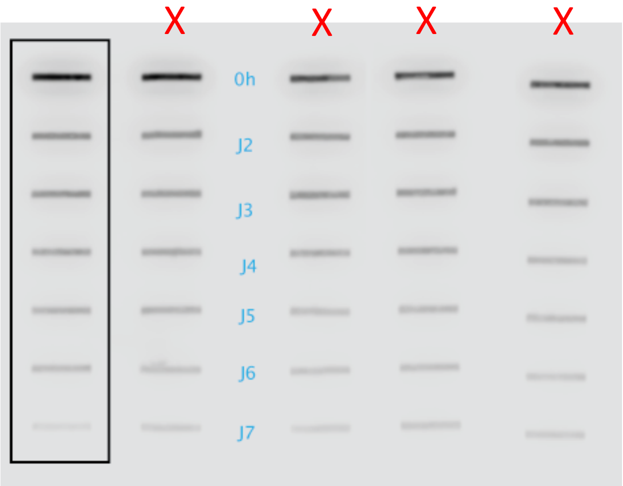

Acute
